# Education Research: Use by Neurologists of Microteaching and Microassessment Programs for Teaching, Learning, and Patient Care Needs: A Qualitative Study

**DOI:** 10.1212/NE9.0000000000200164

**Published:** 2024-11-05

**Authors:** Kara A. Stavros, Alexandra Michelle Miller, Jeremy J. Moeller, Kimberly Wiseman, Sydney-Evelyn Gibbs, Xiaoyu Sun, Lynne Shindoll, Robert Rook, Michaela Morris, Tessa Dahlgren, Zachary London, Roy E. Strowd

**Affiliations:** From the Warren Alpert Medical School of Brown University (K.A.S.), Providence, RI; Memorial Sloan Kettering Cancer Center (A.M.M.), New York, NY; Department of Neurology (J.J.M.), Yale School of Medicine, New Haven, CT; Wake Forest University School of Medicine (K.W., S.-E.G., R.E.S.), Winston-Salem, NC; American Academy of Neurology (X.S., L.S., R.R., M.M., T.D.), Minneapolis, MN; and University of Michigan School of Medicine (Z.L.), Ann Arbor, MI.

## Abstract

**Background and Objectives:**

Microlearning is the acquisition of knowledge or skills in small units, commonly delivered by digital technology. NeuroBytes (NB) and Question of the Day (QOD) are 2 microinstructional programs in neurology. NB programs are brief, video-based mini-courses on clinical topics (microteaching); QODs are daily multiple-choice questions (microassessment). The aim of this study was to understand how neurologists use NB and QOD and to explore their influence on clinical practice, education, and lifelong learning.

**Methods:**

Purposive sampling was used to recruit neurologists or trainees who completed at least 1 NB program or 25 QODs within the past 3 months. Individual semistructured interviews were conducted to explore participants' use of NB/QOD, what they perceived as gained from the product, how learning influenced their practice, and how this influenced lifelong learning. Thematic analysis was conducted to generate codes and describe themes emerging from the data.

**Results:**

A total of 28 participants were interviewed. Neurologists were enthusiastic to use NB and QOD. Three themes were identified. NB and QOD were used to (1) enhance teaching, (2) influence clinical care, and (3) facilitate lifelong learning. Enhancing teaching: Interviewees used knowledge gained from NB/QOD in their own instruction and used NB/QOD with students. QOD was a model for writing their own assessments. Patient care: Respondents kept their knowledge current and reinforced concepts already known. QOD had less impact on patient care because it was used for identifying knowledge gaps, often outside the subspecialty niche. Lifelong learning: QOD was used to identify gaps in knowledge while NB filled educational gaps already recognized. NB and QOD were viewed as timesaving and could be completed on the fly. However, they were considered supplemental to other learning sources because they lacked depth.

**Discussion:**

NB and QOD are convenient supplemental resources for teaching, learning, and augmenting clinical practice. Microteaching and microassessment delivered through these programs fulfilled different learning needs and have complementary educational roles.

## Introduction

Continuing professional development (CPD) is an ongoing process encompassing a range of learning activities that health care professionals must engage with throughout their career trajectory. This includes not only expansion of medical knowledge and clinical skills but also competencies in ethics, interpersonal communication, teaching, professionalism, team building, and management skills.^1^ The methods by which CPD is delivered are widely variable, traditionally ranging from professional conferences to webinars to print journals, among others. As with all medical education, CPD for health care professionals should be inclusive of different learning styles and preferences and evolve with advances in technology and educational innovations. A particular challenge of CPD is that the learning activities must be accommodated within a busy professional practice. Health professionals must either take time off from their primary role to engage in CPD activities (such as conferences or courses) or find time within busy schedules for more limited learning activities.

Microlearning is defined as the acquisition of knowledge or skills in small units, and it is a method for facilitating continuing professional growth and development for health care professionals.^2^ This approach has the benefit of being asynchronous, self-directed, and broadly accessible through electronic devices.^3,4^ The result is that in contrast to more traditional approaches to CPD, which may be lengthier and require investment of time and funds, microlearning allows learners the flexibility to self-determine a more personalized approach to learning and access knowledge spontaneously at any time, in any setting, and for any duration. The short duration of a microlearning activity can help avoid mental fatigue and reduce cognitive load.^5^ Studies demonstrate that microlearning allows learners to remain up to date, builds confidence in clinical procedures, helps retain knowledge over the long term, and increases engagement in collaborative learning.^2,6^

Microlearning may be achieved by microteaching, which is the delivery of educational content in small bursts, or by microassessment, which refers to evaluations completed in small units to assess knowledge and skills.^6^ Microteaching and microassessment are innovative instructional strategies that harness the convenience and appeal of digital technology and are promising resources for CPD for health care professionals. As microteaching and microassessment techniques continue to emerge and develop, there are still outstanding questions regarding their potential uses in the day-to-day practice of health care professionals and their place in an overall CPD framework. Additional clarification is needed regarding the specific type of knowledge and skills that are best suited for microteaching and microassessment vs more traditional formats and whether microlearning meaningfully affects higher level outcomes such as patient care and lifelong learning.^2,6^ Addressing this gap in knowledge will further our understanding of how microlearning can provide a more comprehensive picture of CPD and foster more well-informed employment of these resources. In this study, we aimed to address this gap by examining participants' experiences with 2 American Academy of Neurology (AAN) microlearning programs, NeuroBytes (NB) and Question of the Day (QOD).

## Methods

### Methodologic Approach

This study explored the question of how NB and QOD are used by neurologists in their CPD to identify educational gaps, promote self-directed learning, and inform patient care. To achieve this aim, a qualitative research approach with general qualitative methodology was selected to explore participants' experiences with NB and QOD. We chose a qualitative approach because it would allow us to gain a deeper understanding of the individual experiences of NB and QOD users and help us understand how and why users engaged with these programs. In contrast to typical quantitative evaluation approaches (such as numerical surveys, user ratings, or learning analytics), the use of a qualitative approach in this study helped capture more detailed perspectives and a deeper understanding of how NB and QOD influence clinical, academic, and educational practice.

### AAN Microlearning Products

NB programs are brief (<5 minutes) microteaching videos that focus on clinical topic updates or reviews, using animation and graphics to engage the viewers.^3^ These videos exist in a frequently updated library and can be accessed on a learning management system by members of the AAN using any electronic device (e.g., computer, tablet, and mobile device). QOD is an app-based microassessment program consisting of daily multiple-choice questions with rationales, often supplemented with imaging, with cumulative continuing medical education (CME) credit. NB delivery through the AAN's learning management system is mobile-friendly and allows for tracking progress but requires a dedicated log-in and cannot be pushed directly to mobile devices. By contrast, as an app, the QOD user experience has push technology to allow content to be pushed to users' mobile devices.

The NB and QOD programs were developed by the AAN with the aim of expanding the eLearning portfolio to offer concise and readily available educational opportunities to a large population of learners. Previous research has shown that users find NB to be a user-friendly, easily accessible, and up-to-date resource for neurologists and trainees.^3^ These products were also chosen because of their availability to neurologists through the AAN Online Learning Center, where they can be accessed by all members at no additional cost.

### Participant Sampling and Interview Procedures

A deidentified list was generated of all AAN members who had participated in either NB or QOD in the past year. To select participants who had meaningful engagement with these products, we identified those who had completed at least 1 full NB program or who had completed at least 25 QOD questions and claimed 1 CME credit for QOD participation within the past 3 months. This included students, residents, fellows, and faculty in all practice settings. From this list, a sample of users was sent an email invitation to participate. Purposive sampling was used to balance these email invitations by age, self-identified race, and practice setting. Snowball sampling was used to identify and recruit additional participants until saturation in topics discussed during semistructured interviews was achieved.

Consenting participants were contacted through email and scheduled for one-on-one semistructured interviews with 2 Q-PRO staff members using video conference (Webex, Skype, or FaceTime) or telephone based on participant preference. Each interview lasted approximately 30 minutes and was recorded, transcribed verbatim, and deidentified.

The interview script was developed by a team of experts including medical educators, eLearning experts, and qualitative researchers using the results of an AAN member survey on online learning preferences to guide and inform the development. Interview questions explored when clinicians use NB/QOD, benefits of the teaching, how knowledge gained influences their clinical practice, future needs, and downstream impacts on their approach to learning. For each topic covered, probes were used to explore areas of interest in the research question as they arose during the interviews.

From the initial email invitation, all participants were identified as either a NB or QOD user. This was confirmed in their email response, in the process of scheduling the interview, and at the start of the interview to ensure that interviews were for either NB or QOD specifically. While each individual interview was dedicated to either NB or QOD alone, the same interview script was used for both NB and QOD interviews. This script is available as supplemental material.

### Standard Protocol Approvals, Registrations, and Participant Consents

This study was approved by the institutional review board at Wake Forest University School of Medicine and granted a waiver of the requirements of signed informed consent (IRB# 00070809). Verbal consent was obtained from all participants over the video conference call or telephone call. Consent forms were mailed or emailed and reviewed by all participants before the interviews.

### Data Analysis

NB and QOD interviews were stored and analyzed separately to allow the experiences to be compared. Interviews did not collapse responses across the 2 products, and all comments in an interview were about either NB or QOD. If a participant commented on the product that was not the focus of their interview, this was noted specifically in the interview to maintain this integrity.

All interviews were audio-recorded and transcribed verbatim. The audio was transcribed by an outside company Landmark Associates Inc. Furthermore, the transcript was reviewed for accuracy by listening to the audio while reviewing the transcript line-by-line. Transcripts were imported into Atlas.ti version 9 for data management and analysis.

After reviewing the transcripts, 2 Q-PRO staff members (K.W. and S.-E.G.) developed a draft codebook. The codebook was reviewed by the study team, and edits were made accordingly. K.W. and S.-E.G. then independently coded the transcripts and met regularly to discuss and resolve discrepancies and reach consensus. Once coding was complete, reports were run for each code and data were reviewed within single codes or combinations of codes to identify patterns and themes. Saturation was assessed by monitoring the point at which no new ideas or themes emerged from the data. Saturation was monitored during data collection and assessed during analysis and determined to be achieved.

### Trustworthiness

Trustworthiness in qualitative research demonstrates the consistency and rigor of the data analysis in yielding meaningful results that are reliable and valid. Criteria for trustworthiness include confirmability, credibility, dependability, and reflexivity.^7^ Confirmability and dependability of the data were established by creating a detailed audit trail of coding and thematic analysis, with coding to consensus between the Q-PRO staff members. Credibility was established through triangulation accomplished by data collection from different participant sources. Study transferability was ensured by documenting a detailed description of the participants and scope of the project.

The author team engaged in reflexivity to identify author characteristics as researchers and the impact this may have on the study. The author team consisted of neurologists and neurology medical educators (J.M., Z.L., R.E.S., K.S., A.M.) as well as qualitative research experts (S.-E.G., K.W.) and eLearning experts (L.S., B.R., X.S., T.D., M.M.). The team sought to represent multiple viewpoints with the inclusion of neurologists and non-neurologists as well as authors who are users and nonusers of NB and QOD to allow for a more comprehensive perspective with the data acquisition and analysis. For example, to avoid bias in interpretation of the data by virtue of the topic, qualitative researchers conducted the data analysis rather than eLearning experts and medical educators.

### Data Availability

Anonymized data not published within this article may be made available on request from any qualified investigator.

## Results

Twenty-eight interviews with neurologists were conducted from July 2021 to February 2022. Three interviews were conducted on video conference, and the remainder were completed by telephone. Interviews focused either on the QOD product (QOD; N = 18) or the NB product (NB; N = 10). Participant demographics are given in Table 1. The overall participant median age was 55 years (range 25–77), including 2 retired physicians and 2 residents. Most of the participants (24) were located in the United States. Participants' area of practice included general adult neurology, subspecialty adult neurology, pediatric neurology, and neurohospitalist. For those who shared how long they had been using the NB and QOD products, most had experience using the products for more than 1 year.

**Table 1:** Combined Participant Demographics and Duration of Product Use Reported for NB and QOD

Participant age, y, median (range)	55 (25–77)
Self-identified race/ethnicity	
Non-Hispanic White	11
Hispanic White	5
Asian	4
Black	3
Not reported	5
Practice setting	
Academic	12
Hospital, nonacademic	3
Multispecialty group practice	5
Neurology group practice	3
Solo practice	2
Not reported	3
Area of practice	
General adult neurology	13
Subspecialty adult neurology	9
Pediatric neurology	2
Neurohospitalist	4
Geographic location	
United States	24
International	4
Duration of product use	
Since inception	6
More than 1 year	10
1 year or less	5
Not reported	7

Abbreviations: NB = NeuroBytes; QOD = Question of the Day.

In the first section, we present results detailing participants' user experience with NB and QOD, including participant overall impressions categorized into 3 main themes: enthusiasm, utilization, and suggestions for improvement. In the second section, we describe the impact of NB and QOD on lifelong learning, teaching, and patient care. Third, we identify key similarities and differences in the utilization and benefits of NB and QOD. Illustrative quotations from the interviews are provided in Table 2 and referenced throughout.

**Table 2:** Illustrative Quotations Demonstrating Participants' Perceptions of the Impact of NB and QOD on Patient Care, Teaching, and Lifelong Learning

**Influence on patient care**
1. I learned if there is anything new in the way of either evaluation or management of patients with certain specific or different conditions. It allows me the opportunity to apply that toward my practice. (04-QOD)
2. And so a lot of times I've been able to cite literature that I've heard about through NeuroBytes to my patients, and I feel like it not only makes me more credible, but I'm able to show them that I am up to date on the research and the literature. I have done my research with regards to this topic and I know what I'm talking about to some degree, and so I think that's the way I use it in my clinical practice is explaining to patients why our team is making certain decisions. (25-NB)
3. Well I would say probably indirectly in the sense that there might be a single piece of information that I take away from that that I might incorporate into teaching rounds or in the care of a patient or it simply might serve as confirmation of what I already know, that I'm on the right track in terms of what I'm doing. Even if I don't necessarily have something new, it just helps reinforce an existing behavior in terms of how I'm doing a diagnosis or how I'm treating somebody. (27-NB)
4. It reassures me that, even though I have been off the residency program for so many years, I'm as up to date as the current fellows in residence. Number 2, if there is something new about alternative treatment or an updated treatment, then it reinforces my own learning. (5-QOD)
**Influence on teaching**
5. …when I'm doing conferences for the residents, I remember that, oh, there was a QOD, and this was a bullet from that question. When I'm teaching, suddenly this thought will come to my mind, and I'm able to pass on those words directly to the residents while doing conferences for them. (11-QOD)
6. I've used this as a tool for them and to help teach them… I'll whip out my phone, and we'll do the Question of the Day. I've already done it, of course, because I do it first thing in the morning, but I'll do it again with them. Every neurology resident that I've done it with loves it. (18-QOD)
7. Well, when I have to prepare questions for the exams and take those questions as examples. Not exactly, but I change the answers sometimes. This is like a model. I take these questions as models. (10-QOD)
8. I suppose I would use that strategy as a teacher to emulate that kind of teaching style to focus less on words and probably more on imagery to deliver a concept. (27-NB)
**Influence on lifelong learning**
9. I had hoped to get an idea of where my weaknesses are… It keeps me disciplined to have a broad knowledge base and to continually think about things outside of my own subspecialty. It guards against me having too narrow of a knowledge base… (19-QOD)
10. My area of specialty is so precise. I know a ton about neuroinfectious diseases, but I've always worried that I would be more rusty in regular neurology. It was something that would help me to stay up to date with general neurology or things that I didn't normally see a lot of. (22-NB)
11. I wouldn't rely on it 100 percent, because it's kinda short and it's not really that thorough, but definitely a very, very good supplement… As I see it, NeuroBytes are not there to be the sole principle source of your learning. They're more as a supplement material, and I think that they're really fulfilling their purpose greatly, and especially when it's an interesting case. Then I go and read a paper about it afterwards, and I think that they are doing that really great. I think they're fulfilling their purpose for which they have been made. (01-NB)
12. I think, with other continuing education modalities, people try to fill out the time. Something that I observe with NeuroBytes is that you actually compress a lot of information in just a short period of time, which is very useful. If we do the traditional 1-hour session, very few people are really good storytellers so that they could manage the time appropriately. Most of the time, it gives the impression that they are just adding or taking time just to spend the whole hour or the whole 45 minutes. (12-QOD)

Abbreviations: NB = NeuroBytes; QOD = Question of the Day.

### Participant Experience With NB and QOD

Participants shared their overall impressions of their experiences in using NB and QOD. The themes identified from these impressions include (1) enthusiasm for the products, (2) facilitators of utilization, and (3) suggestions for improvement.

#### Enthusiasm

Many participants shared enthusiasm about the products, especially QOD, and expressed hope that the products will continue to be available. Participants used words/phrases such as “great,” “amazing,” “engaging,” “excited,” and “I'm a big fan.” Participants at every stage of career development expressed that time was a barrier to continuing education and shared satisfaction that QOD and NB reduced the time commitment needed for continuing learning. Participants also expressed that NB and QOD were learning opportunities that are relevant to their practices and fit well with their individual learning styles.

#### Utilization

##### How Participants Were Introduced to These Products

In general, most participants were introduced to these products by colleagues and by the AAN, which are typical means of introduction to new CPD tools in neurology. Most NB users reported being introduced to the videos by colleagues while most QOD users reported being introduced to the QOD app through notification from the AAN.

##### How Participants Used These Products

Participants reported appreciating the “on demand” nature of the products, which allowed them to use the products in a way that best fit their schedules. NB users reported accessing the videos intermittently whenever they had a break in their day but without a set schedule/approach. Many relied on AAN email notifications to remind them to use the product and alert them to new NB videos while others logged in to the AAN website during periodic times they had set aside for research and learning.

By contrast, all QOD users reported accessing the app every day and most QOD users had a set habit for accessing QOD in their daily routine. Participants who had a set routine for QOD reported using QOD either at the start of their morning or late in the day before they go home. The remaining users were less structured about when they accessed the QOD app and often used small breaks in their workday. Most QOD users relied on push notifications on their phones to remind them to use the app.

#### Facilitators of Utilization

Facilitators of utilization reported by participants included the short length of the products, ability to earn CME credit with QOD, and engagement through visual teaching aids and gamification.

##### Length

All users of both products identified the short length as a primary reason why they liked the products, using descriptors such as “brief,” “to the point,” “efficient,” and something that “can be done on the run.” Participants reported that the brevity allows them to incorporate the product into any time in their day and enables more rapid completion compared with other CME tools, aligning with shorter attention spans.

The benefit is it's concise. It goes straight to the point. I really like that. My attention span seems to be decreasing year-by-year [laughter], so the shorter the better. (13-NB)

This is a very good 1-minute deal. You learn something in a minute. You're very busy. If you have time, read journals. If you have 5 minutes, watch the NeuroByte… If you're very tired, QOD is very good—it's a 1-minute thing. You may do one question and you're done. (11-QOD)

A few QOD users discussed that the short length leads to easier retention of the information. Although participants highlighted the brevity of both products, they still indicated that they are effective for delivering a high-level overview of relevant information on a topic.

##### Continuing Medical Education Credit

Many QOD users appreciated that QOD provides CME credit (the NB program is not eligible for CME). QOD users reported that the app makes keeping up with CME certification “easier” and less “cumbersome.” Participants reported that QOD is a preferred way to get CME because it is cost effective, requires less time, requires less preplanning and credit is immediate.

##### Visual Teaching Aid

Most NB users liked the visuals and graphics included in the videos. NB users appreciated the visual aspect of this type of CME because it supports different learning styles, is inclusive of learners who process knowledge with both visual and auditory information, and is generally more engaging for the users.

##### Gamification

Participants also reported that through their participation, learning was made “fun” and “enjoyable.” Users of the QOD app liked how the app had “gamified” continuing learning to make it fun to continue to learn. QOD users also reported liking the metrics provided in the app that they used to monitor their own progress, validate knowledge, and compete against others.

#### Suggestions for Improvement

Participants shared several opportunities for improvement of both the QOD app and the NB videos.

##### Topics Covered by the Products and References

QOD users and NB users differed on their preferred delivery of general and subspecialty topics covered by the products. QOD users felt that there were too many subspecialty topics in the app, especially pediatric questions. However, NB users felt that the content of the videos was too general and would prefer more subspecialty videos. The reason for this difference was unclear and could reflect differences in preference for content delivered by microteaching vs microassessment or preferences related to mode of access by the learning management system vs app.

QOD and NB users consistently mentioned desire for more access to references within the answer sections, and references were a high priority for users across both products.

The other thing that I think would be useful is if, at the end of the explanation, they mention the reference with an official way to access that, let's say, by pressing a link directly. (12-QOD)

##### Technical Issues

Technical issues were rare with NB; however, some QOD participants reported technical issues with the QOD app, such as poor image quality or inability to zoom in on images, reflecting the difference in product platforms.

##### Access

A few NB and QOD users shared a desire to access previous NB videos and QODs. This would allow them to continue learning within the platform and increase ongoing engagement. NB users mentioned that although this is available, it is difficult to use on the AAN website.

### Impact of NB and QOD on Patient Care, Teaching, and Lifelong Learning

#### Patient Care

Many participants reported that QOD and NB affect their patient care (Table 2, quotes 1 and 2). Both users of QOD and NB reported that the products allow them to keep up with new developments in general neurology. Participants indicated that the products boosted their confidence, helped them to be more efficient, and provided reminders of information they may encounter less frequently in practice. For example, in reference to engaging in QOD, 1 participant expressed the following:

When you're in practice, the question comes to mind, “Well, I haven't [done] this in a while. [What] am I missing? Is there new information on this that I need to go look it up?” (2-QOD)

Seeing questions where you think, “Oh, yeah, that's the answer I would have had. Yeah, that's the thing I've known for years,” I think that not only boosts your confidence, but it helps your efficiency so you're not having to go look things up as much. (2-QOD)

Both NB and QOD users also reported using the products to reinforce their clinical knowledge within and outside their subspecialties because they can confirm their current practice and stay up to date on information (Table 2, quotes 3 and 4).

QOD users were less likely to report that the app has had a direct impact on individual patients' care. This is because it was heavily used for identifying gaps in knowledge and maintaining general knowledge, which reflects the microassessment medium. NB users were more likely to report watching videos to increase patient-specific knowledge—some NB users mentioned that they would use NB on topics that were relevant to individual patients. A few NB users even mentioned that they use NB as a tool for teaching patients. For example,

I've been able to cite literature that I've heard about through NeuroBytes to my patients, and I feel like it not only makes me more credible, but I'm able to show them that I am up to date on the research and the literature. I have done my research with regards to this topic and I know what I'm talking about to some degree, and so I think that's the way I use it in my clinical practice is explaining to patients why our team is making certain decisions and because they're backed up with the literature. (25-NB)

#### Teaching

Participants reported that they incorporate the knowledge they learn from QOD and NB into their teaching. The most reported way that QOD and NB have influenced participant's education and teaching was that when they learned new information, they shared that information with their students to enhance learning (Table 2, quote 5). This most often occurred through formal teaching opportunities such as curriculum development, lectures, board review, and rounds. Other participants reported directly reviewing the QOD or NB with their students (Table 2, quote 6). This was more commonly reported for QOD. With QOD, participants reported reviewing the day's question in informal teaching opportunities such as morning discussions. For NB, participants more commonly incorporated specific videos into lectures. Several QOD and NB users reported having recommended to their trainees that they should use the products themselves for self-directed learning.

Unique to QOD, several users reported using the app as a structural model for developing their own assessment items for use with their trainees (Table 2, quote 7). One participant mentioned that QOD has helped them learn how to teach specific content more succinctly. Another participant mentioned that they have begun to emulate the teaching style of NB, focusing more on visuals than text (Table 2, quote 8). One participant reported that this is “Because it helps me to synthesize what I'm teaching, what I am trying to say… What it does is it tells me that you can put education into smaller units more frequently and it's going to be more accepted” (12-QOD).

#### Lifelong Learning

All users reported that QOD and NB have a positive impact on their ongoing learning.

##### Identification of Knowledge Gaps and Blind Spots

QOD users reported using the app to identify gaps in their knowledge, typically outside their subspecialty or for new developments in treatment and pharmacotherapy in the field. Users called these their “weak areas” and “blind spots” (Table 2, quote 9). The QOD users who reported that they did not use the app to identify gaps in their knowledge reported using the app to reinforce their existing knowledge. As a tool for identifying gaps in knowledge, QOD was more likely to spur additional follow-up learning. Users reported the desire to learn more about topics that are new to them and discussed looking up additional information and reading more about areas they identified as gaps.

##### Filling in Knowledge Gaps

By contrast, NB users reported using the videos to fill gaps in knowledge that they have already identified (Table 2, quote 10). They reported reviewing the list of NB topics on the AAN website and choosing to watch specific videos that will address the gaps in their knowledge that they need to fill.

##### Supplemental Learning That Should Be Combined With Other CPD Resources

Participants appreciated the short time commitment and brevity of both QOD and NB but acknowledged that neither could meet their entire needs as ongoing learners. These products are seen as supplemental to other learning because they lack a depth and thoroughness in comparison with other learning opportunities (Table 2, quote 11). When comparing QOD and NB to conferences, lectures, and webinars, participants discussed the value of the brief time investment required by both products vs the significant time investment required to attend a conference or lecture (Table 2, quote 12). By contrast, participants did not express this disparity with the other types of learning they engage in, such as reading journals, UpToDate, and podcasts—primarily because the length of engagement in these is self-directed.

##### Gateway Products That Spur Additional Microlearning

Almost all users reported that they would seek out other microlearning opportunities because this type of learning is “enjoyable,” “easy,” “quick,” “helpful,” “faster and better packaged,” “accessible,” and “in the palm of my hand.” Only a few participants said they would not seek out other microlearning opportunities, some of whom were retired or about to retire.

### Comparison of NB and QOD

Similarities and key differences in participant experiences with NB and QOD outlined in the earlier sections are illustrated in the Figure.

**Figure:**
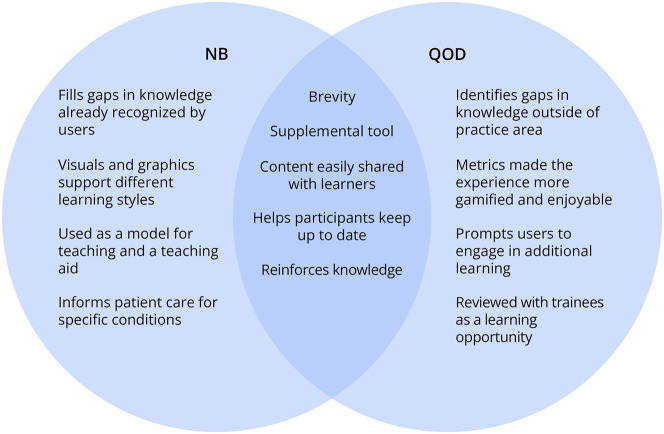
Venn Diagram Illustrating Common Themes and Differences in Participant Experiences With NeuroBytes (NB) and Question of the Day (QOD)

## Discussion

This study addresses a gap in understanding how microlearning influences patient care, lifelong learning, and approaches to teaching for neurologists and trainees. Our results show that (1) NB and QOD facilitate lifelong learning by helping neurologists keep their knowledge current, reinforcing concepts, and fostering a convenient and enjoyable learning experience; (2) NB and QOD influenced patient care with neurologists reporting applying knowledge that was reinforced or gained in clinical scenarios and with some NB participants even using the product to teach patients; and (3) neurologists use NB and QOD as teaching aids and examples for their own educational tools. Despite the overall high satisfaction with the programs, participants identified NB and QOD as supplemental resources that may guide them to pursue additional learning opportunities for deeper understanding of specific topics.

Overall, these findings emphasize the benefits of microlearning as a successful component of CPD. Health care professionals at all stages are interested in using a variety of educational technologies in pursuing CPD, and in the modern learning environment, the most optimal CPD approach must evolve as information technology evolves.^8,9^ Changes in technology and a trend toward personalized, self-motivated, and just-in-time learning reflect the ways that health care professionals learn, socialize, and communicate currently.^10^ The success of microteaching methods has a foundation in appealing to short attention spans and the principle of reducing cognitive load by presenting information in small, manageable units.^11,12^ Furthermore, microteaching and microassessment methods of microlearning can also be easily combined with other learning and teaching strategies. For example, participants using both NB and QOD often shared their knowledge or the products themselves with trainees, adding a blended learning component that incorporates the benefits of social engagement.^13^ For neurology in particular, microlearning may offer an appealing and effective format for CPD that facilitates learning in the face of rapid growth of knowledge in neurology and constraints of time and energy amid a shortage of neurology health professionals. It may also be a useful tool in the ongoing efforts to address neurophobia among students by breaking down knowledge into smaller, more manageable bursts of information.^14^

NB and QOD had many overlapping benefits, but some differences were reported, which may reflect the different advantages derived from microteaching vs microassessment. For example, QOD was more often used to identify knowledge gaps outside the subspecialty area and, therefore, less used in patient care compared with NB. NB was identified as a valuable resource for filling in knowledge gaps already known to participants, making it more applicable to patient care. These findings may suggest that the underlying instructional strategies of microteaching and microassessment can fulfill different learning needs for health care professionals and could serve as complementary tools for individualized, self-directed learning. However, differences may also reflect the format and platforms used because NB was accessed through a learning management system while QOD is an app and mode of utilization may also influence the ultimate benefits of each product. Both microteaching and microassessment have been shown previously to achieve base outcomes in Moore's Level of Outcome framework^15^ for CME, such as participation and knowledge.^2-4^ Our data suggest their value in achieving higher level outcomes as well. Consideration of combining microteaching and microassessment tools may yield even greater benefits for physicians' professional growth and development and could be an avenue for further research.^16^

Study limitations include that only NB and QOD were studied so findings may be limited to these products. It is possible that different findings may be seen with other microlearning products. In addition, NB and QOD are delivered through different platforms, and the opinions and perspectives expressed by the participants could be related to the instructional strategy and the platforms used. It is difficult to disentangle this, although the qualitative approach of probing users' insights is the best strategy to do so. Efforts were made to balance enrollment across different age groups, races, and practice settings and include both trainees and faculty to gain a broad perspective. However, it was not possible to quantify the degree to which various perspectives are shared broadly by the larger neurology community, which is a question that will require further future study to capture a wider range of experiences. In this study, member checking was not performed in analysis of the data and a prolonged and persistent observation of the participants was not possible. The authors engaged in reflexive analysis at every stage of this work, and the interviews, coding, and initial analysis were performed by independent qualitative researchers. However, the authors' personal and professional investment in the success of these tools could introduce a bias of overvaluing the positive elements in participant interviews. We attempted to balance this by giving careful consideration to the perceived limitations of these tools (e.g., focus on superficial learning).

In conclusion, this study suggests that microlearning and microassessment tools such as NB and QOD provide an enjoyable and convenient learning experience that can achieve wide participation and knowledge gains/reinforcement but can also translate into enhanced participant learning, teaching, and patient care. These resources may be best used as introductory or supplementary tools that inspire health care professionals to seek out further learning to expand their knowledge, and they have the flexibility to be used in combination with other learning methods to facilitate continued professional development.

## Appendix Authors

**Table:**

Name	Location	Contribution
Kara A. Stavros, MD	Warren Alpert Medical School of Brown University	Drafting/revision of the manuscript for content, including medical writing for content; analysis or interpretation of data
Alexandra Michelle Miller, MD, PhD	Memorial Sloan Kettering Cancer Center	Drafting/revision of the manuscript for content, including medical writing for content; study concept or design; analysis or interpretation of data
Jeremy J. Moeller, MD, MSc, FRCPC	Department of Neurology, Yale School of Medicine	Drafting/revision of the manuscript for content, including medical writing for content; analysis or interpretation of data
Kimberly Wiseman, MS	Wake Forest University School of Medicine	Drafting/revision of the manuscript for content, including medical writing for content; major role in the acquisition of data; analysis or interpretation of data
Sydney-Evelyn Gibbs, MSGH	Wake Forest University School of Medicine	Drafting/revision of the manuscript for content, including medical writing for content; major role in the acquisition of data; analysis or interpretation of data
Xiaoyu Sun, MSEd	American Academy of Neurology	Drafting/revision of the manuscript for content, including medical writing for content; analysis or interpretation of data
Lynne Shindoll, MA	American Academy of Neurology	Drafting/revision of the manuscript for content, including medical writing for content; analysis or interpretation of data
Robert Rook	American Academy of Neurology	Drafting/revision of the manuscript for content, including medical writing for content; analysis or interpretation of data
Michaela Morris	American Academy of Neurology	Drafting/revision of the manuscript for content, including medical writing for content; analysis or interpretation of data
Tessa Dahlgren	American Academy of Neurology	Drafting/revision of the manuscript for content, including medical writing for content; analysis or interpretation of data
Zachary London, MD	University of Michigan School of Medicine	Drafting/revision of the manuscript for content, including medical writing for content; study concept or design; analysis or interpretation of data
Roy E. Strowd III, MD, MEd, MS	Wake Forest School of Medicine	Drafting/revision of the manuscript for content, including medical writing for content; major role in the acquisition of data; study concept or design; analysis or interpretation of data

## Glossary

AAN: American Academy of Neurology
CME: continuing medical education
CPD: continuing professional development
NB: NeuroBytes
QOD: Question of the Day
